# Draft genome sequence of *Bacillus amyloliquefaciens* HB-26

**DOI:** 10.4056/sigs.4978673

**Published:** 2014-03-15

**Authors:** Xiao-Yan Liu, Yong Min, Kai-Mei Wang, Zhong-Yi Wan, Zhi-Gang Zhang, Chun-Xia Cao, Rong-Hua Zhou, Ai-Bing Jiang, Cui-Jun Liu, Guang-Yang Zhang, Xian-Liang Cheng, Wei Zhang, Zi-Wen Yang

**Affiliations:** 1 National Biopesticide Engineering Technology Research Center, Hubei Biopesticide Engineering Research Center, Hubei Academy of Agricultural Sciences, Wuhan, China; 2 Department of Horticulture, Hubei Vocational College of Bio-technology, Wuhan 430070, China

**Keywords:** *Bacillus amyloliquefaciens* HB-26, The Next-Generation sequencing, *Plasmodiophora brassicae*

## Abstract

*Bacillus amyloliquefaciens* HB-26, a Gram-positive bacterium was isolated from soil in China. SDS-PAGE analysis showed this strain secreted six major protein bands of 65, 60, 55, 34, 25 and 20 kDa. A bioassay of this strain reveals that it shows specific activity against *P. brassicae* and nematode. Here we describe the features of this organism, together with the draft genome sequence and annotation. The 3,989,358 bp long genome (39 contigs) contains 4,001 protein-coding genes and 80 RNA genes.

## Introduction

*Bacillus amyloliquefaciens* (*B. amyloliquefaciens*) is a species of bacterium in the genus *Bacillus* with high affinity of *Bacillus subtilis*. In the growth process, *B. amyloliquefaciens* can produce numerous antimicrobial or, more generally, bioactive metabolites with well-established activity in vitro such as surfactin, iturin and fengycin [1,2]. The production of all of these antibiotic compounds highlights *B. amyloliquefaciens* as a good candidate for the development of biocontrol agents [3,4].

Strain HB-26 belongs to the species *B. amyloliquefaciens*. The type strain of the species produces much bioactive metabolites showing specific activity against *Plasmodiophora brassicae* which could cause Clubroot, one of the most serious diseases of brassica crops worldwide [5-7]. Heavy infection by this pathogen of Chinese cabbage, cabbage, broccoli, turnip, oilseed rape, and other crucifers can lead to severe economic losses [8-11]. The root systems of infected plants show gall formation, which inhibits nutrient and water transport, stunts plant growth, and increases susceptibility to wilting [12,13]. Otherwise, bioassay results showed strain HB-26 also had some root-knot nematicidal activity.

Here, we present a summary classification and a set of features for *B. amyloliquefaciens* HB-26, together with the description of the genomic sequencing and annotation in order to improve the understanding of the molecular basis for its ability to inhibit *Plasmodiophora brassicae* and nematode.

## Classification and features

**Figure 1:**
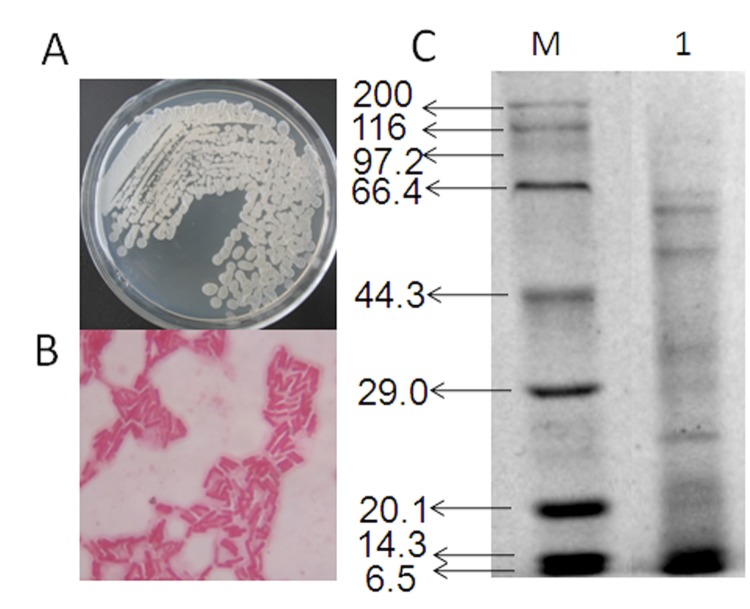
General characteristics of *B. amyloliquefaciens* HB-26. (A) The colonial morphology pictures of strain HB-26. (B) Phase contrast micrograph of HB-26. (C) SDS-PAGE analysis of proteins of HB-26. Lane M, protein molecular weight marker; Lane 1, proteins of strain HB-26.

Strain HB-26 colonies were milky white and matte with a wrinkled surface. Microscopy observations indicated that it was a *Bacillus* species (Figure 1A, Figure 1B and Table 1). SDS-PAGE analysis showed this strain secreted six major protein bands of 65, 60, 55, 34, 25 and 20 kDa (Figure 1C).

**Table 1:** Classification and general features of *B. amyloliquefaciens* HB-26

**MIGS ID**	**Property**	**Term**	**Evidence code**^a^
		Domain *Bacteria*	TAS [14]
		Phylum *Firmicutes*	TAS [15-17]
		Class *Bacilli*	TAS [18,19]
	Current classification	Order *Bacillales*	TAS [20,21]
		Family *Bacillaceae*	TAS [20,22]
		Genus *Bacillus*	TAS [20,23,24]
		Species *Bacillus amyloliquefaciens*	TAS [25-27]
	Gram stain	Gram-positive	NAS
	Cell shape	rod-shaped	IDA
	Motility	mobile	NAS
	Sporulation	Spore-forming	IDA
	Temperature range	Room temperature	NAS
	Optimum temperature	pH7.0	IDS
	Carbon source	organic carbon source	NAS
	Energy source	organic carbon source	NAS
MIGS-6	Habitat	Soil	IDA
MIGS-6.3	Salinity	salt tolerant	NAS
MIGS-22	Oxygen	Aerobic	NAS
MIGS-14	Pathogenicity	Avirulent	NAS
MIGS-4	Geographic location	Hubei, China	IDA
MIGS-4.1	Latitude	30.07N	
MIGS-4.2	Longitude	112.23E	
MIGS-4.3	Depth	5-10cm	
MIGS-4.4	Altitude	about 35m	
MIGS-5	Sample collection time	2009	IDA

a) Evidence codes - IDA: Inferred from Direct Assay; TAS: Traceable Author Statement (i.e., a direct report exists in the literature); NAS: Non-traceable Author Statement (i.e., not directly observed for the living, isolated sample, but based on a generally accepted property for the species, or anecdotal evidence). These evidence codes are from the Gene Ontology project [28].

**Figure 2:**
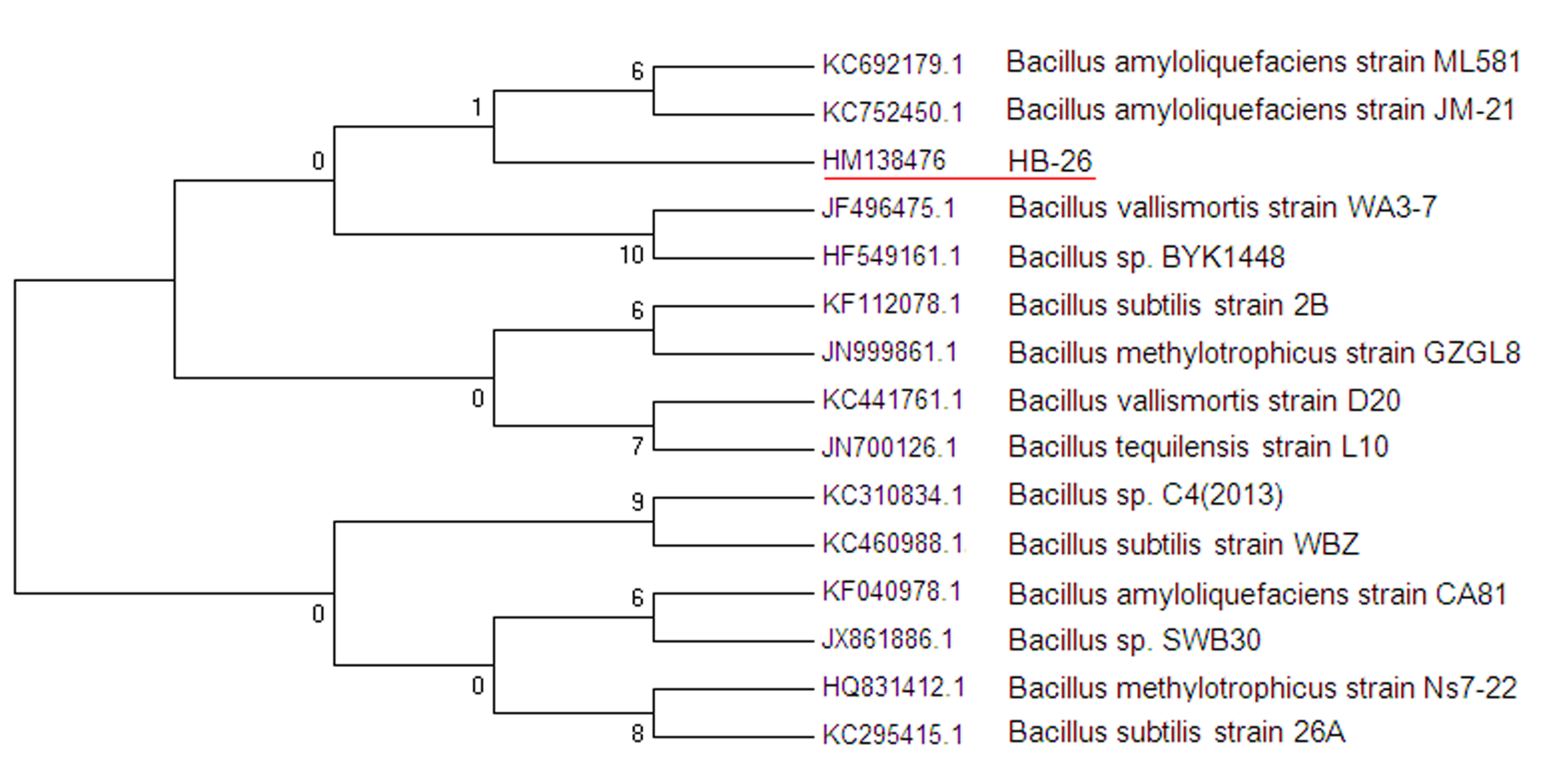
Neighbor-Joining Phylogenetic tree was generated using MEGA 4 based on 16S rRNA sequences. The strains and their corresponding GenBank accession numbers for 16S rDNA sequences are: **A**: *B. amyloliquefaciens* ML581 (KC692179.1); **B**: *B. amyloliquefaciens* JM-21 (KC752450.1); **C**: Bacillus strain HB-26 (HM138476); **D**: *B. vallismortis* WA3-7 (JF496475.1); **E**: *B. sp.*BYK1448 (HF549161.1); **F**: *B. subtilis* 2B (KF112078.1); **G**: *B.methylotrophicus* GZGL8 (JN999861.1); **H**: *B.vallismortis* D20 (KC441761.1); **I**: *B.tequilensis* L10 (JN700126.1); **J**: *B. sp.* C4(2013) (KC310834.1); **K**: *B. subtilis* WBZ (KC460988.1); **L**: *B. Amyloliquefaciens* CA81 (KF040978.1) ; **M**: *B. sp.* SWB30 (JX861886.1) ; **N**: *B.methylotrophicus* Ns7-22 (HQ831412.1); **O**: *B. subtilis* 26A (KC295415.1). The phylogenetic tree was constructed by using the neighbor-joining method within the MEGA software [30].

A representative genomic 16S rDNA sequence of strain HB-26 was searched against GenBank database using BLAST [29]. Sequences showing more than 99% sequence identity to 16S rDNA of HB-26 were selected for phylogentic analysis, and 15 sequences were aligned with ClustalW algorithm. The tree was reconstructed by neighbor-Joining by using Kimura 2-parameter for distance calculation. The phylogenetic tree was assessed by bootstrapped for 1,000 times, and the consensus tree was shown in Figure 2.

## Genome sequencing information

### Genome project history

This *Bacillus* strain was selected for sequencing due to its specific activity against *Plasmodiophora brassicae* and nematode. The complete high quality draft genome sequence is deposited in GenBank. The Beijing Genomics Institute (BGI) performed the sequencing and the NCBI staffs used the Prokaryotic Genome Annotation Pipeline (PGAAP) to complete the annotation. A summary of the project is given in Table 2.

**Table 2:** Genome sequencing project information

MIGS ID	Property	Term
MIGS-31	Finishing quality	Draft
MIGD-28	Libraries used	One genomic libraries, one Illumina paired-end library (700 bp inserted size)
MIGS-29	Sequencing platform	Illumina Hiseq 2000
MIGS-31.2	Sequencing coverage	192 ×
MIGS-30	Assemblers	SOAPdenovo 1.05 version
MIGS-32	Gene calling method	Glimmer and GeneMark
	GenBank Data of Release	August 31, 2016
	NCBI project ID	AUWK00000000
	Project relevance	Agricultural

### Growth conditions and DNA isolation

*B. amyloliquefaciens* HB-26 was grown in 50 mL Luria-Broth for 6 h at 28°C. DNA was isolated by incubating the cells with lysozyme (10 mg/mL) in 2 mL TE (50 mM Tris base, 10 mM EDTA, 20% sucrose, pH8.0) at 4°C for 6 h. 4 mL of 2% SDS were added and the mixture was incubated at 55°C for 30 min; 2 mL 5M NaCl were added, and the mixture was incubated at 4°C for 10 min. DNA was purified by organic extraction and ethanol precipitation.

### Genome sequencing and assembly

The genome of *B. amyloliquefaciens* HB-26 was sequenced using Illumina Hiseq 2000 platform (with a combination of a 251-bp paired-end reads sequencing from a 700-bp genomic library). Reads with average quality scores below Q30 or more than 3 unidentified nucleotides were eliminated. 2,605,589 paired-end reads (achieving ~192 fold coverage [0.94 Gb]) was *de novo* assembled using SOAPdenovo 1.05 version [9]. The assembly consists of 39 contigs arranged in 39 scaffolds with a total size of 3,989,358 bp (including chromosome and plasmids).

### Genome annotation

Genome annotation was completed using the Prokaryotic Genomes Automatic Annotation Pipeline (PGAAP). Briefly, Protein-coding genes were predicted using a combination of GeneMark and Glimmer [31-33]. Ribosomal RNAs were predicted by sequence similarity searching using BLAST against an RNA sequence database and/or using Infernal and Rfam models [34,35]. Transfer RNAs were predicted using tRNAscan-SE [36]. In order to detect missing genes, a complete six-frame translation of the nucleotide sequence was done and predicted proteins (generated above) were masked. All predictions were then searched using BLAST against all proteins from complete microbial genomes. Annotation was based on comparison to protein clusters and on the BLAST results. Conserved domain Database and Cluster of Orthologous Group information is then added to the annotation.

## Genome properties

**Figure 3:**
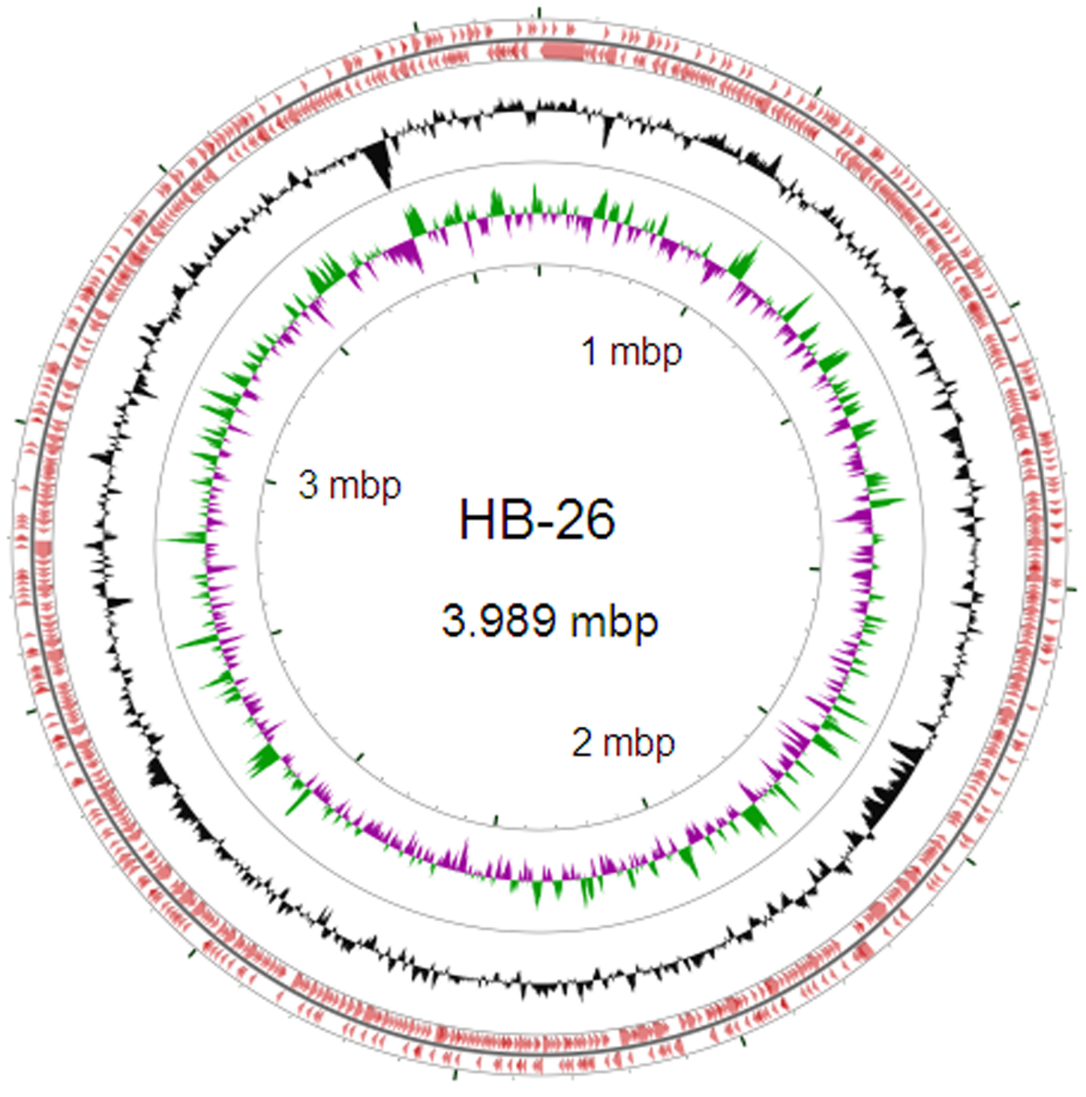
Graphical circular map of the *Bacillus amyloliquefaciens* HB-26 genome. From the outside to the center: genes on forward strand (color by COG categories), genes on reverse strand (color by COG categories), GC content, GC skew. The map was generated with the CGviewer server (Stothard Rearch Group: http://stothard.afns.ualberta.ca/cgview_server/).

The draft assembly of the genome consists of 39 contigs in 39 scaffolds, with an overall 47.37% G+C content. Of the 4,114 genes predicted, 4,001 were protein-coding genes, and 80 RNAs were also identified. The majority of the protein-coding genes (54.06%) were assigned a putative function while the remaining ones were annotated as hypothetical proteins. The distribution of genes into COGs functional categories is presented in Table 3, Table 4 and Figure 3.

**Table 3:** Genome Statistics

**Attribute**	**Value**	**% of total**
Genome size (bp)	3,989,358	100.00
DNA coding region (bp)	3,486,615	87.39
DNA G+C content (bp)	1,889,758	47.37
Number of scaffolds	39	-
Extrachromosomal elements	unknown	-
Total genes	4,114	100.00
tRNA genes	76	1.85
rRNA genes	4	0.1
rRNA operons	0**	-
Protein-coding genes	4,001	97.25
Pseudo gene (Partial genes)	0 (36)	0 (0.87%)
Genes with function prediction (proteins)	2224	54.06%
Genes assigned to COGs	2,336	56.78%
Genes with signal peptides	328	7.97
CRISPR repeats	0	0

**: none of the rRNA operons appears to be complete due to unresolved assembly problems.

**Table 4:** Number of genes associated with the general COG functional categories

Code	Value	% age	Description
J	130	3.160	Translation, ribosomal structure and biogenesis
A	0	0.0	RNA processing and modification
K	262	6.368	Transcription
L	122	2.965	Replication, recombination and repair
B	1	0.024	Chromatin structure and dynamics
D	34	0.826	Cell cycle control, cell division, chromosome partitioning
Y	0	0	Nuclear structure
V	52	1.264	Defense mechanisms
T	153	3.719	Signal transduction mechanisms
M	182	4.424	Cell wall/membrane/envelope biogenesis
N	53	1.288	Cell motility
Z	0	0.000	Cytoskeleton
W	1	0.024	Extracellular structures
U	43	1.045	Intracellular trafficking, secretion, and vesicular transport
O	97	2.358	Posttranslational modification, protein turnover, chaperones
C	177	4.302	Energy production and conversion
G	249	6.053	Carbohydrate transport and metabolism
E	340	8.264	Amino acid transport and metabolism
F	79	1.920	Nucleotide transport and metabolism
H	123	2.990	Coenzyme transport and metabolism
I	117	2.844	Lipid transport and metabolism
P	205	4.983	Inorganic ion transport and metabolism
Q	116	2.820	Secondary metabolites biosynthesis, transport and catabolism
R	435	10.574	General function prediction only
S	287	6.976	Function unknown
	856	20.81	Not in COGs
